# Inoperable poorly differentiated SCC to vertex of scalp progressing into sagittal sinus treated with palliative Cemiplimab

**DOI:** 10.1002/ccr3.5679

**Published:** 2022-08-24

**Authors:** Katie Grounds, O Emmanuel

**Affiliations:** ^1^ Singleton Hospital Swansea Bay University Health Board Port Talbot UK

**Keywords:** dermatology, geriatric medicine, neurosurgery, oncology, pharmacology

## Abstract

Cutaneous squamous cell carcinoma (cSCC) is the second most common non‐melanoma skin cancer after basal cell carcinoma, characterized by the malignant proliferation of epidermal keratinocytes. We report a case of an inoperable cSCC of the scalp extending into the sagittal sinus, now responding well to the monoclonal antibody Cemiplimab.

## Abstract 2

Advanced cutaneous squamous cell carcinoma (cSCC)* is rare, and difficult to treat. Cemiplimab, an antiPD‐1** monoclonal antibody, is the only approved agent for patients with local or metastatic cSCC. We report a unique case of inoperable cSCC of the scalp extending into the sagittal sinus, initially diagnosed as cyst. It reinforces the importance of early diagnosis and the role of cemiplimab as a safe and effective 1st line treatment option.

An 82‐year‐old retired solicitor with a chronic history of unstable sun damaged skin, presented with a 1.5cm lesion on the vertex of his scalp. Clinical and ultrasound evidence demonstrated a retention cyst. The lesion became progressively bigger and ill‐defined, almost now covering 2cm in diameter. Brain CT scan (Figure 1) revealed an ill‐defined mixed density mass in the vertex measuring 2.5 × 7.8 cm on the dura without breaching it, with extensive lytic destruction of the underlying calvarium. An MRI brain (Figure 2) demonstrated destruction of the parietal bone and infiltration of the dura and superior sagittal sinus by the large lesion on the scalp vertex. Further punch biopsies of the tumor and trucut biopsy revealed poorly differentiated squamous cell carcinoma. Patient is currently receiving the biological monoclonal antibody Cemiplimab^1^with good response.

**FIGURE 1:**
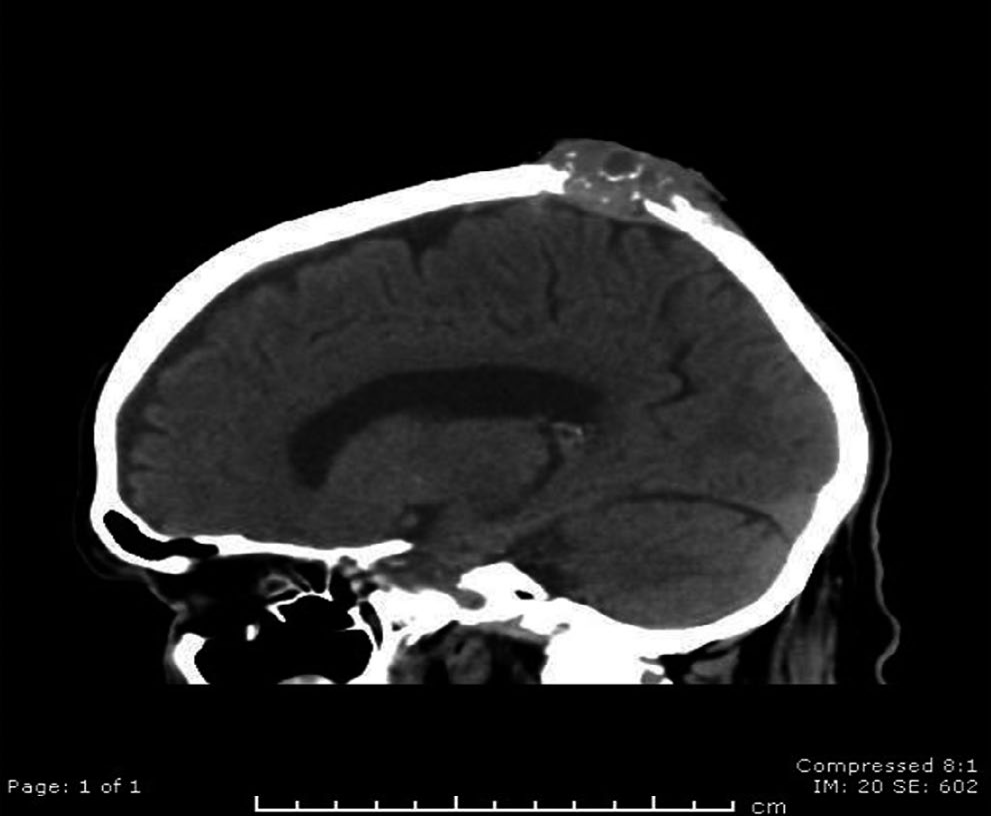
Axial non‐contrast CT image of the skull in bone window showing destructive lesion of the parietal and dura component

**FIGURE 2:**
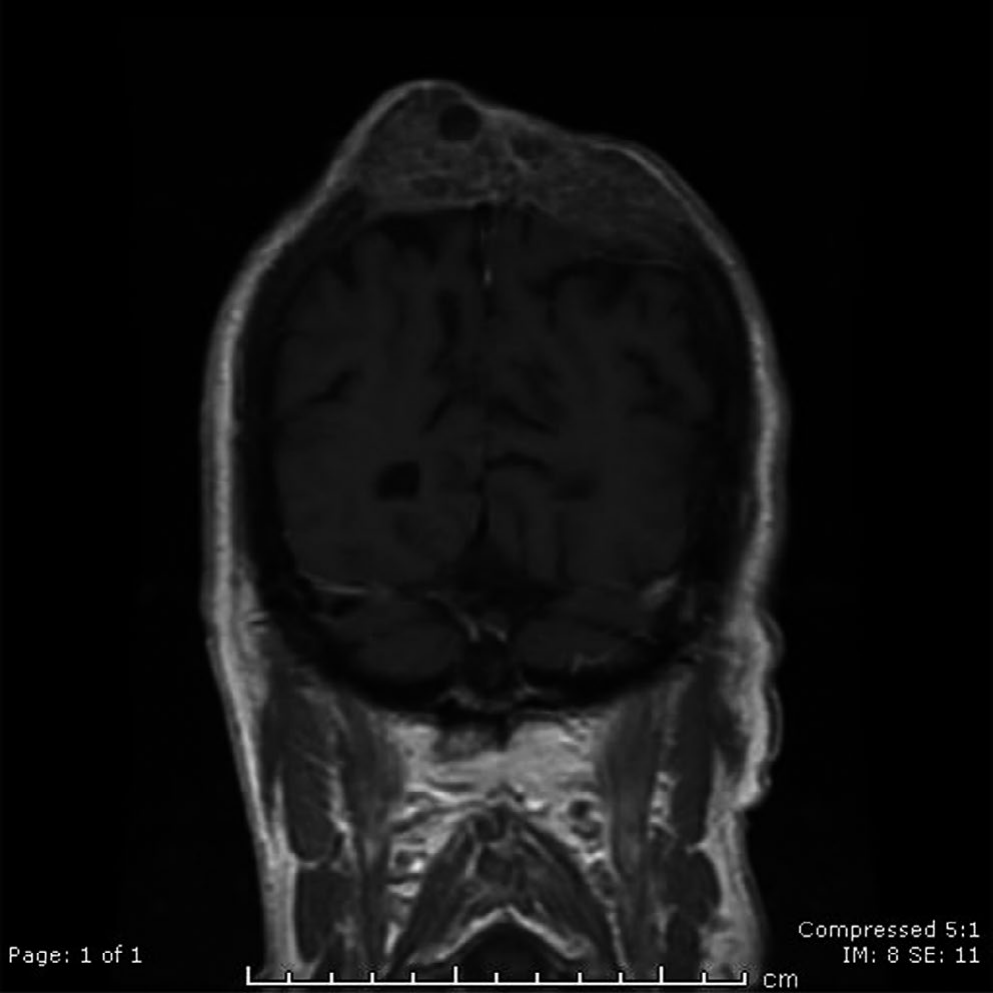
Coronal T1 post‐contrast MRI showing a large destructive, partly cystic mass lesion Involving the parietal bone with non‐homogenous contrast enhancement. *Refers to as an invasive disease, whereby cancer cells have grown beyond the epidermis. ** Programmed cell death protein 1 on the surface of T and B cells in regulation of the immune system's response to cells of the human body

## CONFLICTS OF INTEREST

No conflicts of interest.

## AUTHOR CONTRIBUTION

*Katie Grounds*conceived and designed the analysis, collected and contributed the data, performed the analysis, and wrote the paper. *Odega Emmanuel* conceived and designed the analysis, collected and contributed the data, and performed the analysis.

## CONSENT

Written informed consent was obtained from the patient to publish this report in accordance with the patient's consent policy.

## Data Availability

Data sharing is not applicable.
